# In-depth assessment of acute and chronic house dust mite-induced asthma mouse models

**DOI:** 10.1186/1476-9255-10-S1-P2

**Published:** 2013-08-14

**Authors:** Rachel A Bunting, Josh Wertheimer, Zhao Zhou, Karyn Cochlin, Heather Deutsch, Holly Raymond, Ed Yurkow, Carol Anne Ogden, Ken Kilgore

**Affiliations:** 1 Discovery East Coast Immunology Department, ImmunoPharmacology Group, Janssen Pharmaceuticals, a Johnson & Johnson Company, Radnor PA, USA

## 

Human asthmatic disease is described as a chronic inflammatory disease of the airways. However, the underlying immunological processes involved in asthmatic disease are not fully understood. Animal models have been used to uncover patho-physiologic mechanisms which relate to human disease, leading to discovery and evaluation of therapeutic targets.

We have temporally characterized two models of asthma in mice, an acute and a chronic House Dust Mite (HDM) model to elucidate comparative pathways in each model and ultimately link these to human disease. Specific mechanisms of asthma were evaluated including: mucus overproduction, Th2 cytokine response, airway remodeling, airway constriction, and IgE production.

The acute HDM model in female Balb/c mice is a 10 day consecutive intranasal challenge with house dust mite extract. The model has an early phase cytokine response shown through significant increases of G-CSF, TNFa, IP-10 and KC in the lung on day 1 which could be linked to mechanisms driving inflammation such as chemotaxis of neutrophils and macrophages/monocytes. The height of the inflammatory phase in the acute model is at day 7 with resolution seen by day 16. In contrast, the chronic HDM model is a 5 week model in female Balb/c mice, challenging intranasally 5 times per week with house dust mite extract. The chronic HDM model reveals significant mucus overproduction and airway remodeling characteristics such as significant upregulation of soluble collagen in the lung and tissue inhibitors of metalloproteinase -1 (TIMP-1) which has been shown to be increased in sputum and lung biopsies from asthmatic patients.

By modeling the essential mechanisms involved in asthma through either the acute or chronic HDM model; we will more fully understand human disease and discover superior therapeutics to combat asthma.

